# Draft genome sequence of *Staphylococcus equorum* strain P30 isolated from a fecal specimen of a fermented food-consuming healthy male in Sikkim, India

**DOI:** 10.1128/mra.01329-25

**Published:** 2026-02-18

**Authors:** Srichandan Padhi, Puja Sarkar, Tara Sharma, Arjan Narbad, Amit Kumar Rai

**Affiliations:** 1 BRIC-National Agri-Food and Biomanufacturing Institute (BRIC-NABI) 127373 , Mohali, India; 2 University Centre for Research and Development, Chandigarh University, Mohali, India; 3 Department of Microbiology, STNM Hospital, Gangtok, India; 4 Quadram Institute Bioscience, Norwich, Norfolk, United Kingdom; 5 Department of Botany, University of Delhi 28742, Delhi, India; University of Wisconsin-Madison, Madison, Wisconsin, USA

**Keywords:** *Staphylococcus equorum*, antimicrobial resistance, fermented foods

## Abstract

We report here the draft genome sequence of *Staphylococcus equorum* strain P30, isolated from the feces of a fermented food-consuming healthy volunteer from Sikkim, India. The genome has 2,728,812 bp spread among 57 contigs, with a G+C content of 33%, and contains multiple genes for antimicrobial resistance.

## ANNOUNCEMENT

*Staphylococcus equorum*, originally isolated from healthy horses, is a common and core associate of food microbiota in high salt fermented foods, including cheeses and fermented meat products. Reportedly, this bacterium hydrolyzes the food proteins and lipids to produce flavor compounds, thereby contributing to characteristic sensory profiles (1). However, growing evidence of acquired antimicrobial resistance (AMR) has raised serious concerns for the food industry, emphasizing thorough examination of *S. equorum* strains (2). A *S. equorum* strain was isolated on Brain Heart Infusion (BHI) agar (supplemented with 10 µg/mL vancomycin, 10 µg/mL nystatin, 5 µg/mL cefsulodin, 5 µg/mL trimethoprim, 8 µg/mL amphotericin B, and 2.5 U/mL polymyxin B) from the fecal specimen of a fermented food-consuming healthy male during June 2023 at STNM Hospital, Gangtok, Sikkim. The plates were kept at 37°C for 4–5 days under anaerobic conditions. The isolate was grown in BHI broth at 37°C overnight, and genomic DNA was extracted using the Wizard Genomic DNA Purification Kit (Promega). DNA quantity was measured using Nanodrop 1000 (Thermo Fisher Scientific). The libraries were prepared using the Nextera XT DNA Library Preparation Kit (Illumina) and index primers (Illumina) and quantified using the Promega QuantiFluor dsDNA System (Catalog No. E2670). The final pool was double-SPRI size-selected between 0.5× and 0.7× bead volumes using sample purification beads (Illumina DNA Prep, [M] Tagmentation [96 samples, IPB], 20060059), analyzed with Qubit 3.0, and run on Agilent Tapestation 4200. The pool was run at a final concentration of 1.5 pM on an Illumina Nextseq 500 using a Mid Output Flowcell (NSQ 500 Mid Output KT version 2 [300 cycles], Illumina Catalog FC-404-2003) using a 2 × 150 paired-end protocol (3), generating 1,409,908 reads.

The reads were trimmed and quality checked on Fastp (version 0.23.4, --length_required 36) (4) and FastQC (version 0.12.1) (http://www.bioinformatics.babraham.ac.uk/projects/fastqc), respectively. Genome assembly was performed using SPAdes (version 3.15.5) (5), and the quality of the assembly was measured using QUAST (version 5.0.2) (6). The genome was annotated using the NCBI Prokaryotic Genome Annotation pipeline (7). All bioinformatics analyses were run with predefined settings, unless stated otherwise. Taxonomic assignment of the genome was carried out based on the closest hit, *Staphylococcus equorum* PA231 (NCTC 12414), using Type (Strain) Genome Server (8). Antimicrobial-resistant genes and virulence factors were predicted using AMRprofiler (9) and VFDB 2025 (10). The draft genome has 2,728,812 bp in a total of 57 contigs, with a genome coverage of 52.2381×, G+C content of 33%, *N*_50_ of 293,084 bp, and *L*_50_ of 4. The genome is estimated to have 99.03% completeness and consists of 2,691 genes, including 2,589 protein-coding, 54 tRNA, 5 rRNA (5S, 16S, 23S: 2, 2, 1) genes, and multiple AMR genes (Table 1); however, no virulence factors were detected in the genome.

**TABLE 1:** Antimicrobial resistance genes in the *S. equorum* P30 genome, predicted using AMRprofiler

Resistance gene	Product of the resistance gene	Antibiotic class
dfrG	Trimethoprim-resistant dihydrofolate reductase DfrG	Trimethoprim
mph(C)	Mph(C) family macrolide 2′-phosphotransferase	Macrolide
msr(A)	ABC-F type ribosomal protection protein Msr(A)	Macrolide/streptogramin
mgrA	ATP-binding cassette antibiotic efflux pump; major facilitator superfamily (MFS) antibiotic efflux pump	Cephalosporin; disinfecting agents and antiseptics; fluoroquinolone antibiotic; penam; peptide antibiotic; tetracycline antibiotic
arlR	MFS antibiotic efflux pump	Disinfecting agents and antiseptics; fluoroquinolone antibiotic
dfrC	Trimethoprim-resistant dihydrofolate reductase dfr	Diaminopyrimidine antibiotic
norA	Major facilitator superfamily antibiotic efflux pump	Disinfecting agents and antiseptics; fluoroquinolone antibiotic

## Data Availability

This whole-genome shotgun project has been deposited at DDBJ/ENA/GenBank under the accession JBJIIT000000000. The version described in this paper is version JBJIIT000000000.1. The raw data at the SRA database can be accessed at SRR31344673.
